# Law Regulating the Practice of Dentistry in Georgia

**Published:** 1880-04

**Authors:** 


### EDITORIAL, ETC.

*Law Regulating the Practice of Dentistry in Georgia.*—An act to regulate the practice of dentistry, and to protect the people against empiricism in relation thereto, in the State of Georgia.

SECTION 1.—*Be it enacted by the General Assembly,* That from and after the passage of this Act it shall be unlawful for any person to engage in the practice of dentistry in the State of Georgia, unless said person has graduated and received a diploma from the Faculty of a Dental College, chartered under the authority of some one of the United States or foreign governments, or shall have obtained a license from a Board of Dentists, duly authorized and appointed by this act to issue such license.

SEC. 2.—That the Board of Examiners shall consist of five (5) dental graduates or practitioners of dentistry, who are members in good standing of the Georgia State Dental Society; provided, that said graduates or practitioners have been practicing in the State of Georgia for a term of not less than three (3) years. Said Board shall be elected to serve for two years. The President of said Georgia State Dental Society shall have power to fill all vacancies in said Board for unexpired terms.

SEC. 3.—That it shall be the duty of this Board, first, to meet annually at the time of meeting of the Georgia State Dental Society, or oftener, at the call of any three of the members of said Board. Thirty days notice must be given of the annual meetings. Secondly, to prescribe a course of reading for those who study dentistry under private instructions. Thirdly, to grant a license to any applicant who shall furnish satisfactory evidence of having graduated and received a diploma from any incorporated dental college, without fee, charge or examination. Fourthly, to grant license to all other applicants who undergo a satisfactory examination. Fifthly, to keep a book, in which shall be registered the names of all persons licensed to practice dentistry in the State of Georgia.

SEC. 4.—That the books so kept shall be a book of record, and a transcript from it, certified to by the officer who has it in keeping, with the common seal, shall be evidence in any court in the State.

SEC. 5.—That three members of said Board shall constitute a quorum for the transaction of business, and should a quorum not be present on the day appointed for their meeting, those present may adjourn from day to day until a quorum is present.

Sec. 6.—That one member of said Board may grant a license to an applicant to practice until the next regular meeting of the Board, when he shall report the fact, at which time the temporary license shall expire, but such temporary licenses shall not be granted by a member of the Board after the Board has rejected the applicant.

Sec. 7.—That any person who shall, in violation or this Act, practice dentistry in the State of Georgia for a fee or reward, shall be liable to indictment, and on conviction, shall be fined not less than fifty nor more than three hundred dollars; provided, that nothing in this act shall be construed to prevent any person from extracting teeth; and provided further, that none of the provisions of this Act shall apply to regular licensed physicians and surgeons.

Sec. 8.—That on trial of such indictment, it shall be incumbent on the defendant to show that he has authority, under the law, to practice dentistry, to exempt himself from such penalty.

Sec. 9.—That one-half of all fines collected shall inure to the informer, and the other half to the educational fund of the county.

Sec. 10.—That dentists who have been in practice prior to the passage of this Act, are exempt from all provisions of the same.

Sec. 11.—Repeals conflicting laws.

Approved August 24, 1872. [See Code of Georgia, §1416.]

---

An Act to amend Section 1416 of the Code of Georgia relating to and regulating the Practice of Dentistry in the State of Georgia, and to require Practicing Dentists to register.

Section 1.—*Be it enacted by the General Assembly,* That section 1416 of the Code of Georgia be so amended as to read as follows: "That any person who shall, in violation of this Act, practice dentistry in the State of Georgia for a fee or reward shall be deemed guilty of a misdemeanor, and, upon conviction, shall be punished as prescribed in section 4310 of the Code of 1873; provided, that nothing in this act shall be construed to prevent any person from extracting teeth; and provided further, that none of the provisions of this Act shall apply to regular licensed physicians and surgeons in practice at or prior to the passage of this Act, and dentists who were in practice prior to the 24th of August, 1872."

Sec. 2.—Every person practicing dentistry in this State shall, within sixty days after the passage of this Act, register his name, together with his post-office and the date of his diploma or license, in the office of the Clerk of the Superior Court of the county in which he practices, and shall, on the payment to such Clerk of a fee of fifty cents, be entitled to receive from him a certificate of such registration.

SEC. 3.—That all laws and parts of laws in conflict with this Act be, and the same are hereby repealed.

Approved October 20, 1879.

---

### BIBLIOGRAPHICAL.

